# Homœopathy Going to the Bow-wows

**Published:** 1891-01

**Authors:** 


### HOMŒOPATHY GOING TO THE BOW-WOWS.

I believe in straight outs; no milk-and-water men for me. I have taken pleasure in saying of a neighbor: "He is a homœopath *sans peur sans reproche*," and do not take pleasure in saying "he is a mongrel." And yet I will have to change my method of thinking, for Homœopathy is so greatly changing that in ten years it will be difficult to find the genuine Hahnemannian. Even the *North American Journal of Homœopathy* seems to be on the road to the *bow-wows*. What would an old-fashioned homœopath think of this: "Eminence in symptomatology is certainly commendable and desirable, and doubtless there are *certain minds* especially adapted to make the best use of them. I envy and praise such acquirements, while I deplore universal dependence upon them." This is bad enough, but, shades of Hahnemann, how is this?

"There has always been a noticeable tendency among homœopathic practitioners of therapeutics to disregard in their practice pathology and the careful diagnosis of disease. * * * Aside from the interests of the patients, the practitioner owes it to himself and the profession to carefully consider pathology, lest by increasing ignorance and disregard of it they bring upon themselves, and as far as their influence reaches upon the profession, the opprobrium of being superficial and unscientific."

The last quotation is a good one: "We had at one time an opportunity of watching the practice of a physician who had a large gynæcological clinic. Chronic cases (diseases of uterus) were subjected to active catharsis by the use of purgative pills of the doctor's own make; the patients were directed to report the second day. The first examination might reveal a large subinvoluted, perhaps ulcerated, cervix uteri; on the patient's return we could hardly recognize the case, so great the change brought about by relieving the portal circulation by purgatives. * * * The doctor followed up this advantage by the use of the indicated homœopathic remedy."

These three quotations are from three leading articles in the October number, and by three prominent homœopaths. May we say to these erring brethren, "Drop your distinctive name, your inclination to hang on to the skirts of allopathy, and join the army of eclectics.—*The Eclectic Medical Journal* for December.

['Tis well, sometimes, to hold the mirror up to nature and see ourselves as others see us. THE HOMŒOPATHIC PHYSICIAN has been so constantly the target of malicious shafts aimed at it by those who pretending to be homœopathists are, nevertheless, rank allopathists in their treatment, that we feel justified in shoving the above piquant criticism under their eyes, that they may realize how we are sustained in the logic of our own position by those who differ from us radically.

The very journal that is, in the foregoing article, so sharply criticised, three years since, shot one of its most malignant missiles at us for the very reason that we maintain consistency in theory and practice. The malignant article in question we copied for our readers, and it may be found in vol. VII, page 32. That journal now stands condemned by the men whose views it adopted and methods of practice it imitates.

To get upon a logical plane, it should forthwith drop the word Homœopathy from its title, and join the army of eclectics, agreeably to the advice of *The Eclectic Medical Journal*.—EDS.]

